# ATM and GLUT1-S490 Phosphorylation Regulate GLUT1 Mediated Transport in Skeletal Muscle

**DOI:** 10.1371/journal.pone.0066027

**Published:** 2013-06-11

**Authors:** Stanley Andrisse, Gaytri D. Patel, Joseph E. Chen, Andrea M. Webber, Larry D. Spears, Rikki M. Koehler, Rona M. Robinson-Hill, James K. Ching, Imju Jeong, Jonathan S. Fisher

**Affiliations:** Department of Biology, Saint Louis University, St. Louis, Missouri, United States of America; Universitat de Barcelona, Spain

## Abstract

**Objective:**

The glucose and dehydroascorbic acid (DHA) transporter GLUT1 contains a phosphorylation site, S490, for ataxia telangiectasia mutated (ATM). The objective of this study was to determine whether ATM and GLUT1-S490 regulate GLUT1.

**Research Design and Methods:**

L6 myoblasts and mouse skeletal muscles were used to study the effects of ATM inhibition, ATM activation, and S490 mutation on GLUT1 localization, trafficking, and transport activity.

**Results:**

In myoblasts, inhibition of ATM significantly diminished cell surface GLUT1, glucose and DHA transport, GLUT1 externalization, and association of GLUT1 with G_α_-interacting protein-interacting protein, C-terminus (GIPC1), which has been implicated in recycling of endosomal proteins. In contrast, ATM activation by doxorubicin (DXR) increased DHA transport, cell surface GLUT1, and the GLUT1/GIPC1 association. S490A mutation decreased glucose and DHA transport, cell surface GLUT1, and interaction of GLUT1 with GIPC1, while S490D mutation increased transport, cell surface GLUT1, and the GLUT1/GIPC1 interaction. ATM dysfunction or ATM inhibition reduced DHA transport in extensor digitorum longus (EDL) muscles and decreased glucose transport in EDL and soleus. In contrast, DXR increased DHA transport in EDL.

**Conclusions:**

These results provide evidence that ATM and GLUT1-S490 promote cell surface GLUT1 and GLUT1-mediated transport in skeletal muscle associated with upregulation of the GLUT1/GIPC1 interaction.

## Introduction

Impaired insulin-stimulated glucose transport by glucose transporter 4 (GLUT4) is a well-documented contributor to the reduced glucose clearance found in subjects with type 2 diabetes mellitus (T2DM) [1]–[4]. However, subjects with T2DM also display deficiencies in basal glucose transport and lowered GLUT1 protein levels in skeletal muscle [5]. Unlike GLUT4, which is found primarily in skeletal muscle, heart, and adipose tissue, GLUT1 is present in all tissue types [6]. GLUT1 is reportedly responsible for about 30–40% of basal glucose uptake in skeletal muscle, with GLUT4 mediating the balance of basal glucose uptake [7], [8]. Furthermore, GLUT1 is a prominent transporter of dehydroascorbic acid (DHA) [9], [10], the oxidized form of ascorbic acid. GLUT3 and GLUT4 also display DHA transport activity, though the Km for DHA transport is higher for GLUT3 than it is for GLUT4 or GLUT1 [10], [11].

Recent studies have shown that the carboxy terminal of GLUT1 is a key regulator of GLUT1 subcellular localization, trafficking, and activity [12]–[15]. In skeletal muscle, GLUT1 is mainly localized to the plasma membrane. In contrast, GLUT4 is for the most part intracellularly localized under basal conditions. However, a chimeric GLUT4 with a GLUT1 c-terminal is localized to the plasma membrane [12], [16]. Intriguingly, truncation or mutation of the c-terminal PDZ binding motif of GLUT1 resulted in intracellular localization [15]. Likewise, decreased G_α_-interacting protein-interacting protein, C-terminus (GIPC1), a PDZ binding protein, resulted in a reduction of cell surface GLUT1 in epithelial cells [14]. In clone 9 cells, stomatin (STOM) was shown to decrease GLUT1-mediated glucose transport by interacting with the GLUT1 c-terminus [17]. Collectively, these studies show the importance of GLUT1’s c-terminal in its overall regulation, involving interactions of GLUT1 with GIPC1 or STOM.

ATM is a phosphatidylinositol-3-kinase (PI3K) family serine/threonine protein kinase that has been shown to play a role in regulation of glucose transport in cultured cells [18], [19]. Furthermore, transgenic mice expressing non-functional ATM are hyperglycemic [20], [21], underlining a role of ATM in glucoregulation. In addition, skeletal muscles of rats with induced insulin resistance via high fat diet feeding displayed lowered ATM protein levels [19] suggesting a role of ATM deficiency in the development of T2DM. Although the c-terminal of GLUT1 contains a known ATM target, S490 [22], the roles of ATM and GLUT1-S490 in GLUT1 regulation have yet to be elucidated. The goal of the current study was to test the hypothesis that GLUT1-mediated transport activity and plasma membrane localization are regulated by ATM and GLUT1-S490 in skeletal muscle.

## Research Design and Methods

### Materials

Dulbecco’s modified Eagle’s medium (DMEM), phosphate buffered saline, and trypsin were purchased from Sigma Aldrich (St. Louis, MO). The radiolabeled chemicals, ^3^H-2-deoxyglucose, ^14^C-mannitol, and ^14^C-ascorbic acid, were purchased from American Radiolabeled Chemicals, Inc. (St. Louis, MO). Antibodies against phosphorylated ATM substrates and GAPDH were purchased from Cell Signaling Technology, Inc. (Danvers, MA). The anti-FLAG and anti-tubulin antibodies were purchased from Sigma-Aldrich Corp. (St. Louis, MO). The anti-stomatin antibody was purchased from Abnova (Jhongli City, Taiwan). The anti-GIPC1 antibody was purchased from Thermo Scientific (Waltham, MA). The GLUT1 antibody was a generous gift from Michael Mueckler of Washington University (St. Louis, MO). Doxorubicin was purchased from Sigma-Aldrich (St. Louis, MO). The ATM inhibitor KU55933 (KU) [23] was a gift from KuDos Pharmaceuticals, Ltd. (Cambridge, UK). A second ATM inhibitor, CP466722 (CP) [24], was a gift from Pfizer (Groton, CT).

### Animals

Mice that were heterozygous for a truncation mutation of ATM that codes for ATM protein lacking the catalytic domain [25] were obtained from The Jackson Laboratory (Bar Harbor, ME) and used to set up a breeding colony. Animals used in experiments were homozygous for the mutation or were wild-type littermates from this colony. Genotyping was performed from tail snips as previously described [25]. Surgeries were performed using sodium pentobarbital (mg/kg) anesthesia. Precautions to reduce animal suffering were taken.

### Ethics Statement

All procedures using live animals were approved by Saint Louis University’s Institutional Animal Care and Use Committee (Permit number: A-3225-01; Association for Assessment and Accreditation of Laboratory Animal Care accreditation date: 6/18//2009).

### Cell Culture

L6 myoblasts, a cell line derived from rat skeletal muscle, were obtained from the American Type Culture Collection (Manassas, VA). L6 myoblasts were cultured in DMEM containing 10% FetalPlex (Gemini Bio-Products, Woodland, CA) and 1% antibiotic-antimycotic solution at 37°C in 5% CO_2_. For some experiments, myoblasts were differentiated into myotubes in DMEM containing 2% horse serum.

### GLUT1 Constructs and Transfections

The FLAG-GLUT1 construct [26], which contains a FLAG epitope in the first extracellular loop, was graciously provided by Jeffrey Rathmell (Duke University). Site mutants S490A and S490D were generated using the Agilent QuikChange Site Directed Mutagenesis Kit (Stratagene) and sequenced at the Washington University Protein and Nucleic Acid Chemistry Laboratory (PNACL). Plasmids were amplified in DH5α *Escherichia coli* and purified using Qiagen Miniprep Kits. L6 myoblasts, having increased transfection efficiency and lowered GLUT3 expression compared to myotubes [27], were used to facilitate transfections. L6 myoblasts were grown to 80–90% confluency and transfected with using Lipofectamine 2000 in Opti-MEM medium (Sigma-Aldrich, St. Louis, MO) with 1.6 µg/ml of FLAG-GLUT1 plasmid. After 4 hours, transfection medium was replaced with fresh DMEM. Transfected cells were assayed 48 hours after transfections.

### Western Blot Analysis

L6 myoblasts were transiently transfected with FLAG-GLUT1 24–48 hours prior to experiments, serum starved for 3 hours in serum free media, washed with ice-cold PBS, incubated with 6 µM CP for 30 minutes or 1 µM DXR for 60 min, washed with ice-cold PBS, and scraped in lysis buffer (50 mM HEPES pH 7.4, 150 mM NaCl, 10% glycerol, 1% Triton X-100, 1.5 mM MgCl, 1 mM EDTA, 10 mM NaPO_4_, 100 mM NaF, 2 mM NaVO_4_, 10 µg/ml leupeptin, 0.5 µg/ml pepstatin, 10 µg/ml aprotinin, and 1 mM phenylmethylsulfonyl fluoride). Cell lysates were centrifuged at 13,000 rpm for 10 minutes at 4°C, and supernatants were assayed for protein content by the bicinchoninic acid (BCA) method. Samples in Laemmli sample buffer plus dithiothreitol were run on 4–20% pre-cast polyacrylamide electrophoresis gels (Thermo Scientific, Waltham, MA) and transferred to nitrocellulose membranes. For analyses of ATM and P-ATM, samples were run on 3–8% Tris-acetate gels (Invitrogen). The membranes were then blocked with 5% nonfat milk in Tris-buffered saline plus Tween (TBST), incubated in primary antibodies, incubated with horseradish peroxidase-conjugated secondary antibodies, and then detected using enhanced chemiluminescence (PerkinElmer Life Sciences, Boston, MA). Bands were quantified with TotalLab software (Nonlinear Dynamics, Newcastle, UK).

### GLUT1 Localization/Trafficking Assays

The localization and trafficking assays performed were adapted from Wijesekara et al [28] and Ishikura et al [29] with the exception that L6 myoblasts transiently expressing FLAG-GLUT1 were used instead of L6-GLUT4*myc* cells. For determination of cell surface FLAG-GLUT1, cells were incubated on ice with anti-FLAG antibodies (1 hour), incubated with horseradish peroxidase-linked secondary antibodies, and subjected to spectrophotometric analysis at 492 nm with *o*-phenylenediamine as the substrate for generation of yellow-orange product. For the GLUT1 internalization assay, cells were incubated on ice with anti-FLAG antibodies, and then cell surface GLUT1 levels were assessed periodically after re-warming the cells to 37°C. For assessment of GLUT1 externalization, cells were allowed to internalize GLUT1/antibody complexes for two hours at 37°C, and then GLUT1 cell surface levels were evaluated over time after incubation at room temperature to facilitate externalization.

### Immunoprecipitation Assays

L6 myoblasts transiently transfected with FLAG-GLUT1 constructs were treated (plus/minus ATM inhibitor/activator) for one hour. Cell lysates were incubated with FLAG antibodies overnight at 4°C, and then protein A sepharose beads were added to the mixture and incubated for 4 hours at 4°C. The samples were microcentrifuged, washed five times with lysis buffer, and resuspended with 4x Laemmli sample buffer plus dithiothreitol. The samples were then subjected to SDS-PAGE western blot analysis.

### Transport Assays for Cultured Myoblasts

L6 myoblasts were serum starved for 3 hours, incubated in HEPES-Buffered Saline (HBS: 5 mM glucose, 20 mM HEPES, 140 mM NaCl, 5 mM KCl, 2.5 mM MgSO_4_, 1 mM CaCl_2_) for 30 min in the presence or absence of ATM inhibitors/activators, washed several times with glucose-free HBS, and exposed to 2-deoxy-glucose (2DG) transport media (3 µCi/ml 3H-labeled 2DG, 10 µM 2DG, dissolved in glucose-free HBS) or DHA transport media (1 µCi/ml 14C-ascorbic acid, 2 U/ml ascorbate oxidase, and 200 µM DHA, dissolved in glucose-free HBS) [9] for 10 min. The cells were then washed with an ice-cold 0.9% saline solution, incubated with lysis buffer (0.2% SDS and 0.2 N NaOH) for 30 min, and processed for scintillation counting. Samples were subjected to a BCA assay to determine protein content.

### Transport Assays for Skeletal Muscle

Transport in skeletal muscle was studied as described previously [30], [31]. *S*odium pentobarbital (50 mg/kg) was used to anesthetize the animals. Leg muscles were excised and then incubated in Krebs Henseleit bicarbonate buffer (KHB) (32 mM mannitol, 8 mM glucose, and 0.1% radio-immunoassay grade bovine serum albumin (BSA) for one hour. *Glucose transport.* Muscles were washed with KHB containing 40 mM mannitol and 0.1% BSA for 10 minutes at 30°C, incubated in 2DG transport media (KHB with 0.1% BSA, 4 mM 2DG, 3 µCi/ml 3H-2DG, and 0.2 µCi/ml 14C-mannitol) for 10 minutes at 30°C, blotted, and stored at −80°C for further analysis. 14C-Mannitol was used to measure extracellular space. All incubations contained 100 µM indinavir to inhibit GLUT4 activity [8], [32], thus making this assay specific for GLUT1-mediated transport. *DHA transport.* Muscles were incubated in DHA transport media (KHB with 0.1% BSA, 1 µCi/ml 14C-ascorbic acid, 2 U/ml ascorbate oxidase, and 200 µM DHA) for 10 min at 30°C, washed twice with a 200 µM DHA rinse (KHB with 0.1% BSA, no radiolabel) for 5 minutes on ice (in order to prevent DHA efflux), blotted, and stored at −80°C for further analysis.

### Glucose and DHA Transport Studies

In order to account for non-specific uptake, a subset of the muscles and cells were incubated in the presence of 10 µM cytochalasin B. Cytochalasin B inhibits transport by GLUTs. Glucose and DHA transport rates in myoblasts and in skeletal muscle were adjusted by subtraction of the transport rates found for myoblasts or muscles incubated in the presence of cytochalasin B. *Muscle processing.* Muscle samples were ground in lysis buffer (described above) and spun for 10 minutes at 13,000 RPM. Sample supernatant was used for scintillation counts. BCA protein assays were performed to determine protein concentrations. *Scintillation counting. S*ample or incubation media was added to scintillation vials containing Ultima Gold scintillation fluid (PerkinElmer, Waltham, MA). Samples were processed using the Tri-Carb 3110 TR Liquid Scintillation Analyzer (PerkinElmer, Waltham, MA).

### Statistical Analyses

Findings were analyzed using univariate one-way ANOVA with significance determined as p<0.05.

## Results

### ATM Inhibition Diminishes Basal Glucose Transport

Glucose transport in skeletal muscle is predominantly mediated by GLUT1 and GLUT4, with GLUT1 being responsible for physiologically-relevant portion of basal glucose transport [33]–[35]. To determine the effects of ATM inhibition on basal glucose transport, we incubated L6 myotubes in the presence or absence of ATM inhibitors and then assayed glucose transport. ATM inhibition with 1 µM KU or 6 µM CP reduced basal glucose transport by about 50% (DMSO 1.00±0.18, KU 0.52±0.09; DMSO 1.00±0.13, CP 0.38±0.07; p<0.05 for KU and CP respectively).

### ATM Inhibition Diminishes GLUT1-mediated Transport and Reduces GLUT1 Cell Surface Localization

**Figure 1:**
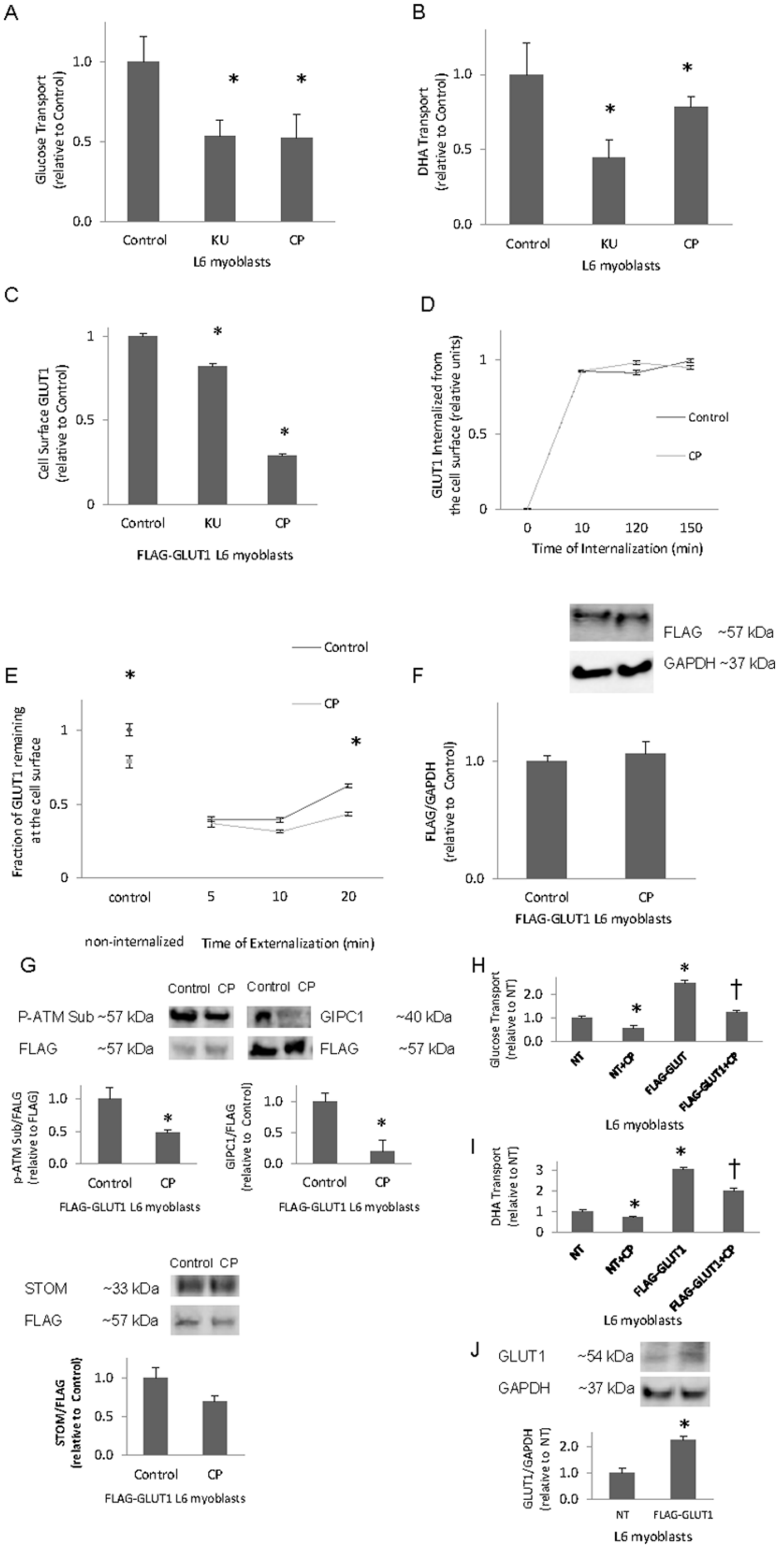
ATM inhibition reduces cell surface GLUT1, GLUT1-mediated transport, ATM phosphorylation of GLUT1 and GLUT1-GIPC1 association. **A)** L6 myoblasts were subjected to a glucose transport assay in the presence of GLUT4 inhibitor, indinavir, and in the presence and absence (0.1% DMSO vehicle) of ATM inhibitor, KU55933 (KU) at 10 µM or CP466722 (CP) at 6 µM, for one hour (n = 9/group); Or **B)** DHA transport assay in the presence or absence (0.1% DMSO) of ATM inhibitors, KU and CP at 10 µM and 6 µM, respectively (n = 9/group). **C)** L6 myoblasts transiently transfected with FLAG-GLUT1 constructs were incubated in the presence or absence of ATM inhibitors, KU and CP, 10 µM and 6 µM, respectively, for one hour, then put through a GLUT1 cell surface assay. (n = 18/group) **D)** FLAG-GLUT1 L6 myoblasts were incubated in the presence or absence of ATM inhibitor, CP, at 6 µM for one hour, and subjected to a GLUT1 internalization assay (n = 12/group), **E)** subjected to a GLUT1 externalization assay. (n = 6/group), or **F)** subjected to Western blot analysis probing for FLAG or glyceraldehyde 3-phosphate dehydrogenase (GAPDH) (n = 4). **G)** Transiently transfected FLAG-GLUT1 L6 myoblasts exposed to 6 µM CP or DMSO (vehicle) for one hour were subjected to immunoprecipitation with antibodies against FLAG. Immunoprecipitates were probed with antibodies against FLAG, phosphorylated substrates of ATM (P-ATM Sub, n = 3), G_α_-interacting protein-interacting protein, C-terminus (GIPC1, n = 6), and stomatin (STOM, n = 6). H) Non-transfected NT and FLAG-GLUT1 transfected L6 myoblasts incubated plus or minus 6 µM CP for one hour were subjected to a glucose transport assay (n = 6/group), Or I) a DHA transport assay (n = 6/group), both in the presence of indinavir. J) GLUT1 and GAPDH levels in non-transfected or FLAG-GLUT1-transfected myoblasts (n = 6/group). For panels H and I: †p<0.05 relative to FLAG-GLUT1. For all panels *p<0.05 relative to NT.

To follow up on the results described above, we determined the effects of ATM inhibition on glucose and DHA transport, GLUT1 localization, and GLUT1 trafficking. Myoblasts, having reduced GLUT3 and GLUT4 levels compared to myotubes [27], [36], were used for these studies instead of myotubes to facilitate transfection procedures. Transport assays were performed in the presence of a GLUT4 inhibitor, indinavir [8], [32]. Indinavir reduces basal glucose transport in L6 myoblasts by 31% (Control 1.00±0.09, indinavir 0.69±0.05; p<0.05). As described above for myotubes, KU or CP diminished glucose transport (by 46% and 48%, respectively (p<0.05, Fig. 1A). Thus, the indinavir-induced decreased basal glucose transport was further diminished by ATM inhibition. Likewise, KU or CP reduced DHA transport (55% and 25%, respectively; p<0.05) (Fig. 1B). In summary, acute inhibition of ATM impairs glucose and DHA transport.

L6 myoblasts transfected with an exo-facially tagged FLAG-GLUT1 construct and treated with ATM inhibitors KU and CP exhibited decreased GLUT1 cell surface localization (18% and 72%, respectively; p<0.05) (Fig. 1C). The differences observed between the effects of KU and CP on cell surface GLUT1 and DHA transport could be attributable to off-target effects of the inhibitors or the effective concentrations of the inhibitors. Alternatively, the effect size of each individual measurement could be subject to experiment-to-experiment variation. Regardless, the qualitative effects of the two inhibitors are the same, in that both inhibitors decrease cell surface GLUT1, glucose transport, and DHA transport.

To discover the means by which ATM inhibition causes a reduction of membrane-localized GLUT1, L6 myoblasts were subjected to GLUT1 internalization and externalization assays and Western analyses for GLUT1 protein levels. CP-treated myoblasts displayed the greatest reduction in cell surface GLUT1 and therefore were used for the subsequent studies. Nearly all cell surface GLUT1 was internalized by 10 minutes in the absence or presence of the ATM inhibitor, CP. The fraction of GLUT1 remaining at the cell surface was the same for ATM inhibitor treated and control cells (Fig. 1D). Therefore, ATM inhibition had no effect on internalization of GLUT1 in L6 myoblasts. In contrast, by 10 minutes, L6 myoblasts treated with 6 µM CP displayed a reduced rate of GLUT1 externalization (21% reduction, p<0.05; Fig. 1E). By 20 minutes, the externalization of GLUT1 between the basal and ATM inhibited L6 myoblasts was reduced still further (31%; p<0.05, Fig. 1E) which may partially account for the depleted cell surface GLUT1 seen in figure 1C. FLAG-GLUT1 protein levels were unaltered by ATM inhibition (Fig. 1F). Thus, inhibition of ATM interferes with recycling of GLUT1 to the cell surface. It should be noted that only exogenous/transfected GLUT1 containing FLAG is assayed by the cell surface, internalization, externalization, and Western analysis assays.

### ATM Inhibition Reduces GLUT1 Phosphorylation and Interaction with GIPC1

The carboxy terminus of GLUT1 contains a PDZ binding motif which includes a known (S490) ATM phosphorylation site [22]. Previous studies have shown that GIPC1 promotes cell surface GLUT1 by associating with the GLUT1 PDZ binding motif [14], [15]. Stomatin, an integral membrane protein, has been shown to decrease GLUT1-mediated glucose transport via a physical interaction with its carboxy terminus [17]. To determine the effects of ATM inhibition on the phosphorylation of GLUT1 and the interaction of GIPC1 or STOM with GLUT1, a FLAG immunoprecipitation assay with the cell lysates from FLAG-tagged GLUT1 L6 myoblasts exposed to 6 µM CP was performed. As shown in figure 1G, CP caused a 52% (p<0.05) decrease in the reactivity of FLAG immunoprecipitates with antibodies against phosphorylated ATM substrates (P-ATM Sub). The P-ATM Sub bands coincided precisely with FLAG-GLUT1, suggesting that GLUT1 is an ATM substrate and that CP decreases phosphorylation of GLUT1 by ATM. CP reduced the amount of co-immunoprecipitated GIPC1 compared to vehicle-treated FLAG-GLUT1 myoblasts by 30% (p<0.05; Fig. 1G). The stomatin/GLUT1 interaction was not statistically different in control vs. CP treated samples (Fig. 1G). Thus, it appears that decreased reactivity of FLAG-GLUT1 immunoprecipitates with the antibody against phosphorylated ATM substrates is associated with decreased GIPC1 co-immunoprecipitation.

L6 cells express GLUT4 and GLUT3 in addition to GLUT1. While the role of GLUT4 has been minimized by the use of indinavir, it remains possible that GLUT3 could mediate some of the changes observed in glucose or DHA transport. If GLUT3 were to have a substantial role in basal glucose transport and DHA transport in the myoblasts, and a treatment (e.g. CP) were to affect transport specifically by modulation of GLUT3-mediated transport, then cells transfected with FLAG-GLUT1 would have a lesser sensitivity to CP in regard to transport when compared with non-transfected cells. However, as shown in Fig. 1H–I, this is not the case. This suggests that GLUT1-mediated transport, as opposed to GLUT3-mediated transport, is the primary mode of transport affected by CP in these assays done in the presence of the GLUT4 inhibitor indinavir. As shown in figure 1J, cells transfected with FLAG-GLUT1 have more than a two-fold greater level of GLUT1 than nontransfected cells (p<0.05), which is roughly equivalent to the fold increase of glucose and DHA transport in transfected cells shown in fig. 1H–I.

### Augmentation of GLUT1-mediated Transport and GLUT1 Cell Surface Localization by ATM Activation

**Figure 2:**
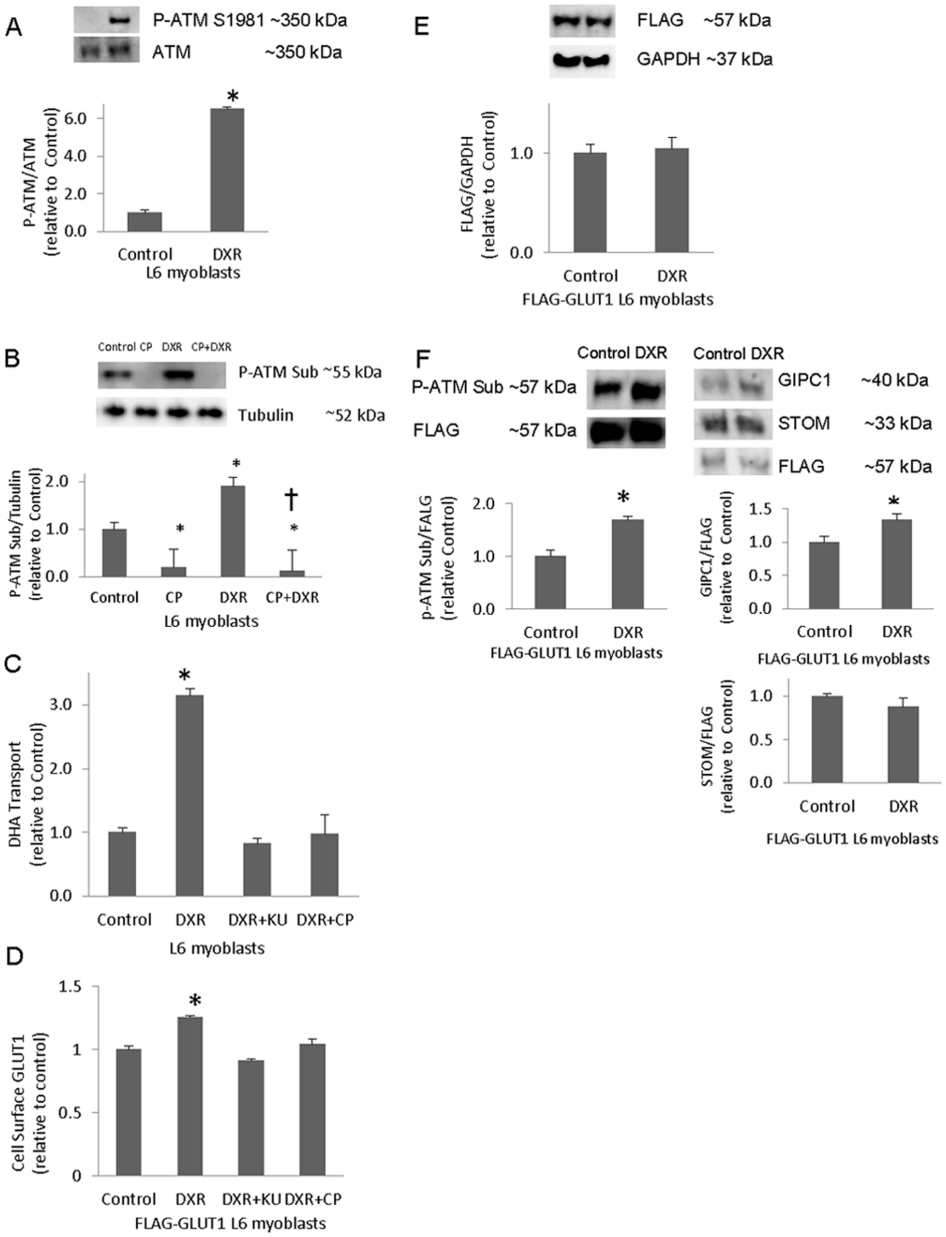
ATM activation enhances GLUT1 cell surface localization, GLUT1-mediated transport, and the GLUT1/GIPC1 interaction. L6 myoblasts were incubated with 1 µM doxorubicin (DXR), 6 mM CP, CP and DXR in tandem, or vehicle for 1 h before western analysis of A) phosphorylated ATM S1981 (P-ATM) and total ATM (n = 3/group) or B) phosphorylated ATM substrates (P-ATM Sub) and tubulin (n = 3–6/group) (*p<0.05 compared to Control; †p<0.05 compared to DXR). C) L6 myoblasts were subjected to a DHA transport assay in the presence or absence (0.1% DMSO vehicle) of 1 µM DXR or 1 µM DXR in tandem with 10 µM KU or 6 µM CP for one hour (n = 9/group). **D)** FLAG-GLUT1 L6 myoblasts were incubated in the presence or absence of 1 µM DXR or 1 µM DXR in tandem with 10 µM KU or 6 µM CP for one hour, then subjected to an assay for cell surface GLUT1 (n = 6/group). **E)** FLAG-GLUT1 L6 myoblasts were incubated in the presence or absence of ATM activator, DXR, at 1 µM for one hour and subjected to Western blot analysis probing for FLAG and glyceraldehyde 3-phosphate dehydrogenase (GAPDH) (n = 4). **F)** Transiently transfected FLAG-GLUT1 L6 myoblasts incubated with 1 µM DXR or vehicle for one hour were lysed and immunoprecipitated with FLAG antibodies. Immunoprecipitates were probed for, phosphorylated substrates of ATM (P-ATM Sub, n = 3), GIPC1 (n = 6), stomatin (STOM, n = 6), and FLAG. For all panels *p<0.05.

We hypothesized that activation of ATM would lead to augmented GLUT1 transport activity and increased cell surface GLUT1. To address this hypothesis we incubated myoblasts with the ATM activator, doxorubicin (DXR). DXR has been shown to increase phosphorylated S1981 ATM (P-ATM) levels in mouse embryonic fibroblasts (MEF) [37]. ATM phosphorylation at S1981, its autophosphorylation site, is a marker of its kinase activity. Here, we show that DXR induces a 6-fold increase in P-ATM S1981 compared to total ATM in L6 myoblasts (p<0.05, Fig. 2A).

To assess the specificity of the P-ATM Sub antibody, whole cell lysates from L6 myoblasts that were treated plus or minus 6 µM CP and/or 1 µM DXR underwent Western blot analysis probing for P-ATM Sub. Because GLUT1 has a molecular weight of 55 kiloDaltons (kDa), we focused on the P-ATM Sub signal around this molecular weight. CP decreased P-ATM Sub reactivity at the band adjacent to the 55 kilodalton (kDa) molecular weight marker (MWM) (p<0.05, Fig. 2B). DXR increased P-ATM Sub at the 55 kDa MWM band (p<0.05), while CP prevented the increase in P-ATM Sub stimulated by DXR (p<0.05). Sensitivity of the P-ATM Sub band to activation (DXR) or inhibition (CP) of ATM suggests that the band is an ATM target.

DXR caused a 3-fold increase in DHA transport (p<0.05, Fig. 2C). The DXR-induced augmentation of DHA transport was prevented by ATM inhibitors, KU or CP (Fig. 2C), suggesting the specificity of ATM’s involvement in DHA transport in response to DXR. DXR enhanced GLUT1 cell surface localization (26%; p<0.05, Fig. 2D), while KU or CP attenuated the DXR-induced increase in cell surface GLUT1. FLAG-GLUT1 protein levels were not statistically changed by ATM activation (Fig. 2E). Interestingly, the DXR-induced increase in cell surface GLUT1 was relatively small compared to the 3-fold increase of DHA transport. This suggests that the DXR effect on DHA transport involves an increase in intrinsic transporter activity.

### ATM Activation Promotes GLUT1 Phosphorylation and Interaction with GIPC1

The ATM activator DXR increased FLAG-immunoprecipitate reactivity with phosphorylated ATM substrate antibodies that corresponded directly with the FLAG-GLUT1 band, suggesting that GLUT1 is an in vivo ATM substrate. DXR also augmented GLUT1-associated GIPC1 by 34% (p<0.05; Fig. 2F). The GLUT1/stomatin interaction was not statistically altered by DXR (Fig. 2F). Therefore, activation of ATM appears to increase GLUT1 phosphorylation and the association of GLUT1 with GIPC1.

### Regulation of GLUT1-mediated Transport and Cell Surface GLUT1 by GLUT1-S490A and S490D Point Mutations

Previous studies have indicated the importance of the GLUT1 carboxy terminus in the regulation of the transporter [12], [13]. Truncation of the last four amino acids on GLUT1 results in diminished cell surface GLUT1 [15]. S490, a known ATM kinase target, resides within the last four residues of GLUT1. Above, we have determined that ATM plays a role in GLUT1 regulation. To determine whether S490 plays a role in GLUT1 regulation, FLAG-GLUT1 S490 was point mutated to alanine (S490A), thus preventing phosphorylation at that residue, or aspartic acid (S490D), mimicking phosphorylation at S490.

**Figure 3:**
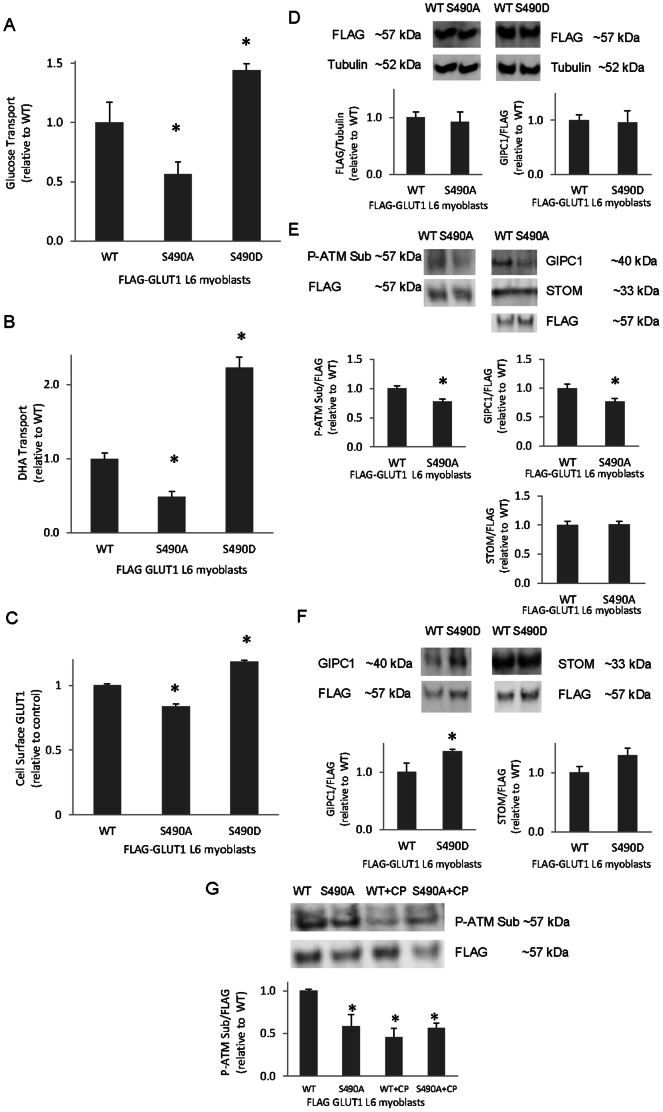
GLUT1-S490 point mutations regulate cell surface GLUT1, glucose and DHA transport, and the GLUT1/GIPC1 interaction. **A)** L6 myoblasts transiently transfected with FLAG-GLUT1 or FLAG-GLUT1 with S490A or S490D point mutations were subjected to a glucose transport assay in the presence of GLUT4 inhibitor, indinavir (n = 9/group) or **B)** a DHA transport assay (n = 9/group). **C)** FLAG-GLUT1 S490A and S490D L6 myoblasts were subjected to a GLUT1 cell surface assay. (n = 6/group). **D)** FLAG-GLUT1 and FLAG-GLUT1 S490A and S490D L6 myoblasts were harvested and subjected to Western blot analysis probing for FLAG and tubulin (n = 3). **E)** Transiently transfected FLAG-GLUT1 and FLAG-GLUT1 S490A or **F)** FLAG-GLUT1 S490D L6 myoblasts were lysed and immunoprecipitated with FLAG antibodies. Immunoprecipitates were probed for FLAG, phosphorylated substrates of ATM (P-ATM Sub (E only), n = 3), GIPC1 (n = 6), and stomatin (STOM, n = 3). G) FLAG-GLUT1 and FLAG-GLUT1 S490A L6 myoblasts lysates were immunoprecipitated with anti-FLAG then probed for P-ATM Sub and FLAG (n = 3/group). For all panels, *p<0.05.

To examine the role of GLUT1 S490 in GLUT1 transport, a glucose transport assay was performed in the presence of GLUT4 inhibitor, indinavir. As shown in figure 3A, L6 myoblasts expressing FLAG-GLUT1 S490A displayed reduced glucose transport (44%; p<0.05). In contrast, S490D mutants exhibited enhanced glucose transport (44%; p<0.05, Fig. 3A). Furthermore, FLAG-GLUT1 S490A mutants displayed diminished DHA transport, while S490D mutants showed increased DHA transport compared to controls (52% and 2-fold, respectively; p<0.05, Fig. 3B). FLAG-GLUT1 S490A L6 myoblasts exhibited a decline in GLUT1 cell surface localization (17%; p<0.05, Fig. 3C), while FLAG-GLUT1 S490D mutants demonstrated a rise in cell surface GLUT1 (19% respectively; p<0.05, Fig. 3C). S490A and S490D mutations had no significant effect on FLAG-GLUT1 levels (Fig. 3D). The S490A- induced decrease in glucose transport correlates well with the ATM inhibition-induced decrease in glucose uptake but is more prominent than the S490A-induced decrease seen in cell surface GLUT1. This suggests that S490 may be a residue involved in regulating GLUT1 intrinsic activity.

### GLUT1-S490A and -S490D Point Mutations Alter GLUT1 Reactivity with Antibodies Against Phosphorylated ATM Substrate and the Interaction of GLUT1 with GIPC1

FLAG immunoprecipitates from L6 myoblasts expressing FLAG-GLUT1-S490A demonstrated decreased reactivity with antibodies against phosphorylated ATM substrates, corresponding directly with the FLAG-GLUT1 band (Fig. 3E). The GLUT1-S490A mutation reduced the GLUT1/GIPC1 interaction by 23% compared to wild-type FLAG-GLUT1 (p<0.05, Fig. 3E) whereas the S490D mutation augmented the GLUT1/GIPC1 interaction (36%; p<0.05, Fig. 3F). The GLUT1/stomatin interaction was unchanged by the S490A and S490D mutations (Fig. 3E-F). Taken together, the data suggest that both ATM and GLUT1 S490 help regulate the association of GLUT1 with GIPC1.

In Fig. 3E, reactivity of the FLAG immunoprecipitate with the antibodies against phosphorylated ATM substrates was not completely prevented by expression of the FLAG-S490A mutant. The c-terminus of GLUT1 contains a known ATM phosphorylation site, S490 [22], and a phosphorylation site, S473, that matches the S/T-Q ATM target motif (www.phosphosite.org) [38]. Both sites receive similar prediction scores for phosphorylation by ATM (NetPhosK) [39]. The incomplete decrease in the P-ATM substrate signal in the FLAG-GLUT1 S490A mutants could potentially be due to phosphorylation of S473.

As shown in Fig. 3E, expression of the FLAG-GLUT1-S490A mutant did not completely abolish coimmunoprecipitation of GIPC1. GLUT1 is known to form functional dimers and tetramers [40]. It is therefore possible that endogenous GLUT1 could associate with both GIPC1 and FLAG-GLUT1-S490A, thus mediating coimmunoprecipitation of GIPC1 with the FLAG construct. If this is the case, the S490A mutation might not fully eliminate the coimmunoprecipitation of GIPC1.

To determine whether ATM inhibition decreases the phosphorylation of GLUT1-S490, wild-type and GLUT1-S490A L6 myoblasts incubated in DMSO or 6 µM CP were harvested, immunoprecipitated with antibodies against FLAG, and then subjected to Western Blot analysis. As shown in fig. 3G, presence of CP or expression of the S490A mutation both decreased reactivity of FLAG immunoprecipitates with the P-ATM Sub antibody.

### Effects of Chronic ATM Deficiency or Acute ATM Inhibition on GLUT1-mediated Transport and GLUT1 Protein Levels in Skeletal Muscle

**Figure 4:**
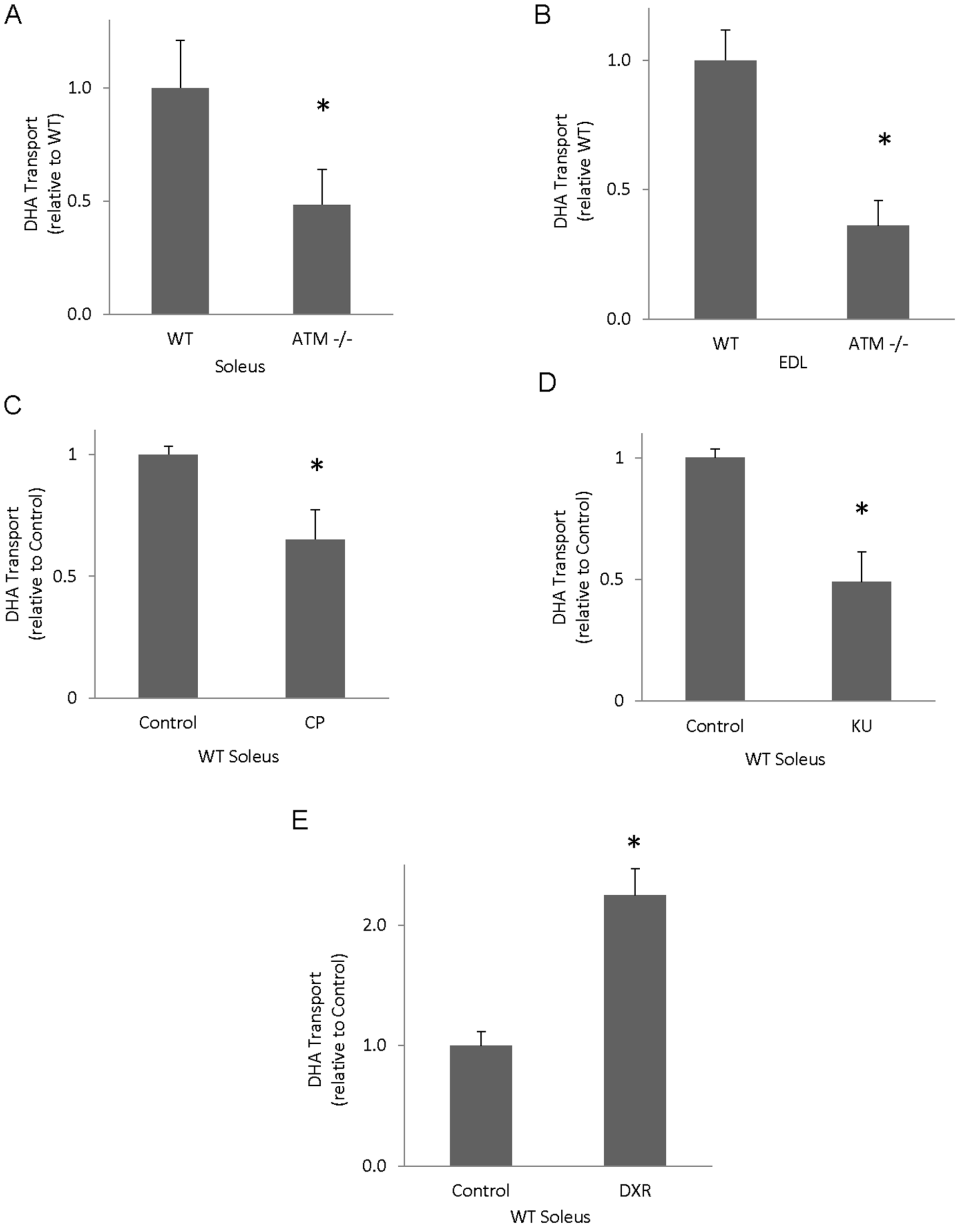
Effects of chronic ATM deficiency or ATM inhibition on GLUT1-mediated DHA transport in skeletal muscle. Wild type (WT) and ATM mutant (ATM −/−) mouse muscles were excised and subjected to a DHA transport assay in the presence of indinavir, a GLUT4 inhibitor. **A)** WT versus mutant soleus muscles (n = 10–12/group). **B)** WT versus mutant extensor digitorum longus (EDL) (n = 8–10/group) **C)** Wild type soleus muscles plus/minus 6 µM CP, **D)** 10 µM KU (n = 5–10/group), or **E)** 1 µM DXR (n = 5–6/group). For all panels, *p<0.05.

The data presented thus far have shown that acute ATM inhibition or activation affects GLUT1 regulation in cultured muscle cells. To determine the effects of chronic ATM loss on GLUT1-mediated transport, glucose and DHA transport studies were performed using a transgenic mouse line expressing truncated, non-functional ATM. Cytochalasin B, which eliminates GLUT-mediated transport, essentially completely prevented DHA and glucose uptake in skeletal muscle (data not shown). ATM mutant soleus and extensor digitorum longus (EDL) muscles exhibited diminished DHA transport compared to wild type control muscles (55% and 65%, respectively; p<0.05, Fig. 4A–B) suggesting that ATM plays a role in DHA transport in skeletal muscle.

To further assess the role of ATM in the regulation of GLUT1-mediated DHA transport activity, wild type mouse muscles were subjected to DHA and glucose transport assays in the presence of indinavir, a GLUT4 inhibitor. Thus, with GLUT4 inhibited, DHA and glucose transport is attributable to GLUT1. Soleus muscles exposed to 6 µM CP or 10 µM KU displayed reduced DHA transport (35% and 54%, respectively; p<0.05, Fig. 4C–D). In contrast, soleus muscles exposed to DXR had 2-fold greater DHA transport than vehicle-treated controls (p<0.05, Fig. 4E). DXR treated EDL muscles displayed a non-significant increase in DHA transport (Control 1.00±0.36, DXR 1.57±0.41). To our knowledge, these are the first data showing DHA transport into skeletal muscle. These data suggest that acute inhibition or activation of ATM regulates DHA transport in intact skeletal muscle.

**Figure 5:**
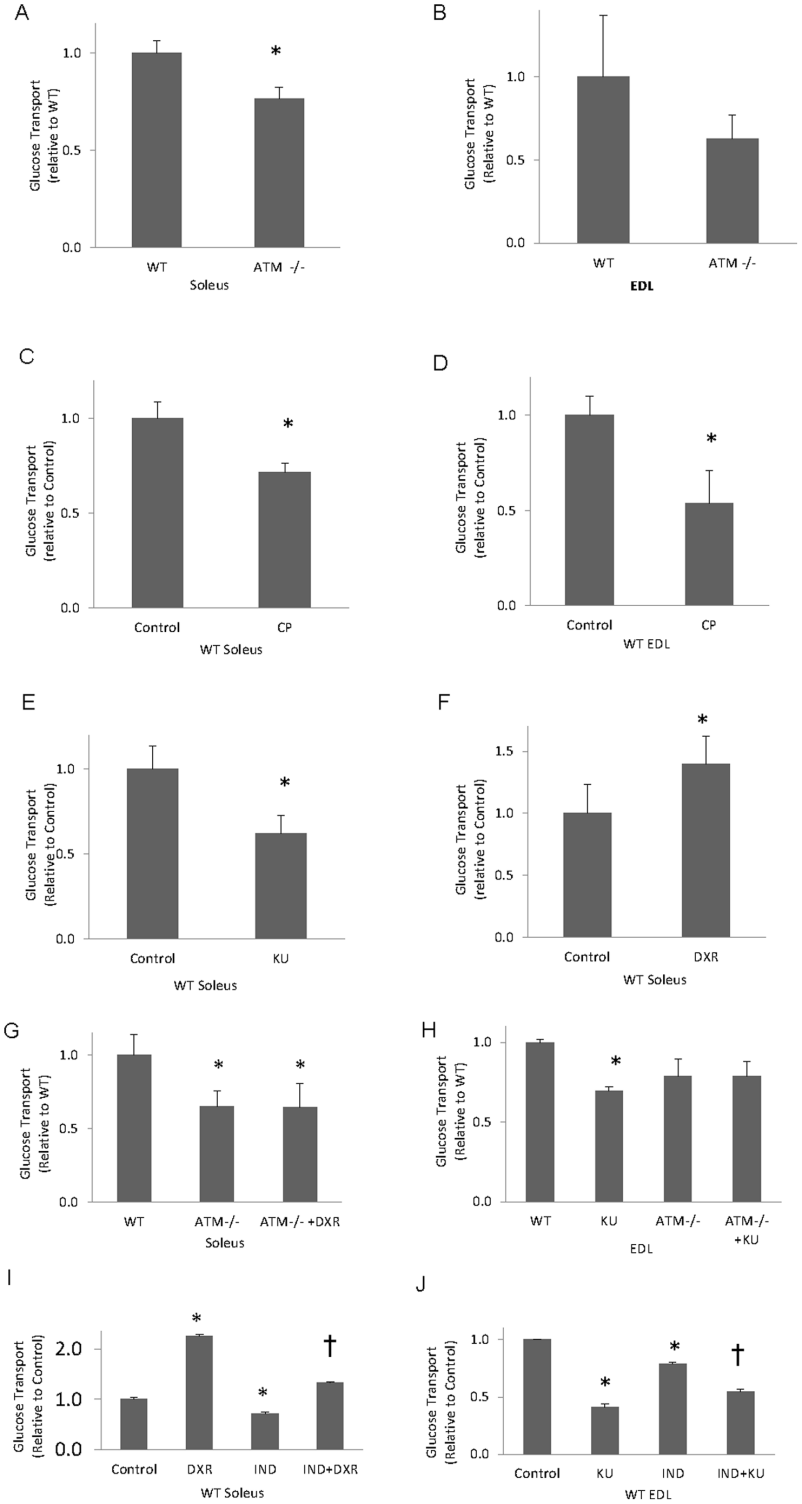
The effects of chronic ATM deficiency and acute ATM inhibition on GLUT1-mediated glucose transport in skeletal muscle. Wild type (WT) and ATM mutant (ATM −/−) mouse muscles were excised and subjected to a glucose transport assay in the presence of indinavir, a GLUT4 inhibitor. **A)** WT versus mutant soleus muscles (n = 8/group). **B)** WT versus mutant EDL muscles (n = 6/group) **C)** Wild type soleus muscles plus/minus 6 µM CP (n = 5/group). **D)** WT EDL muscles plus/minus 6 µM CP (n = 5/group). **E)** WT soleus muscles plus/minus 10 µM KU (n = 6/group). **F)** WT soleus muscles plus/minus DXR (n = 7/group). G) ATM−/− soleus muscles plus/minus DXR relative to WT (n = 3/group). H) WT and ATM−/− EDL muscles in the presence or absence of KU (n = 3/group). I) WT soleus muscles plus/minus 1 µM DXR and in the presence or absence of indinavir (IND) (n = 3/group). J) WT EDL muscles plus/minus 10 µM KU and in the presence or absence of IND (n = 3/group). For panels I and J: †p<0.05 relative to IND. For all panels, *p<0.05.

ATM mutant soleus muscles displayed reduced glucose transport (20%; p<0.05, Fig. 5A), which, given the inhibition of GLUT4 by indinavir, is attributable to GLUT1-mediated transport. ATM mutant EDL muscles displayed a non-significant decrease in glucose transport (Fig. 5B). Soleus and EDL muscles incubated in 6 µM CP and soleus muscles exposed to 10 µM KU demonstrated reduced glucose transport (28%, 46% and 39%, respectively; p<0.05, Fig. 5C–E). In contrast, soleus muscles exposed to the ATM activator, DXR, exhibited an increase in glucose transport (42%; p<0.05, Fig. 5F).

As shown in Fig. 5G, the ATM activator DXR did not affect glucose transport in soleus from ATM−/− mice. Furthermore, ATM inhibition by CP decreased glucose transport in EDL from wild-type mice but had no effect on EDL from ATM−/− animals (Fig. 5H). These data suggest that DXR and CP effects on glucose transport are mediated by their actions on ATM [41].

ATM has been shown to play a role in GLUT4-mediated glucose transport [18], [19], [41]. To determine the effects of ATM inhibitors and activators in the absence and presence of indinavir, glucose transport was assessed in skeletal muscle for the crossed condition of presence or absence of indinavir and presence and absence of ATM inhibitor/activator. As shown in Table 1, indinavir decreases basal glucose transport by about 40% in soleus and EDL. Thus, GLUT1 is responsible for a substantial portion of basal glucose transport. CP inhibits glucose transport both in the absence (49–53%) and in the presence (29–35%) of indinavir. Doxorubicin causes a 2-fold increase in glucose transport both in the absence and presence of indinavir (p<0.05; Fig. 5I). Relative effects of indinavir and KU in EDL are similar to the effects of CP discussed above (p<0.05; Fig. 5J). Thus, the ATM inhibitors and activator are likely to influence both GLUT4-mediated glucose transport and GLUT1-mediated transport. However, the mechanisms for changes in glucose transport in the presence of ATM inhibitors or activators probably rely on Akt- or AS160-mediated effects for the indinavir sensitive portion of glucose transport [18], [19], [41] and, as the data in the current study suggest, direct effects on GLUT1 for the indinavir-insensitive portion of glucose transport.

**Table 1:** Glucose transport in skeletal muscle.

	absence of IND	presence of IND	IND effect
**Soleus**			
–CP	2.44±0.86	1.47±0.04	−40.0%
+CP	1.25±0.11	1.18±0.08	
effect of CP	−48.8%	−29.0%	
**EDL**			
−CP	2.13±0.34	1.35±0.24	−36.6%
+CP	1.00±0.18	0.87±0.12	
effect of CP	−53.1%	−35.6%	

Soleus and extensor digitorum (EDL) longus muscles were incubated in the absence or presence of indinavir (IND) and then in the absence or presence of the ATM inhibitor CP466722 (CP). Values are means and standard errors (n = 3).

* indicates a CP effect (p<0.5), and.

† indicates an indinavir effect (P<0.05).

**Figure 6:**
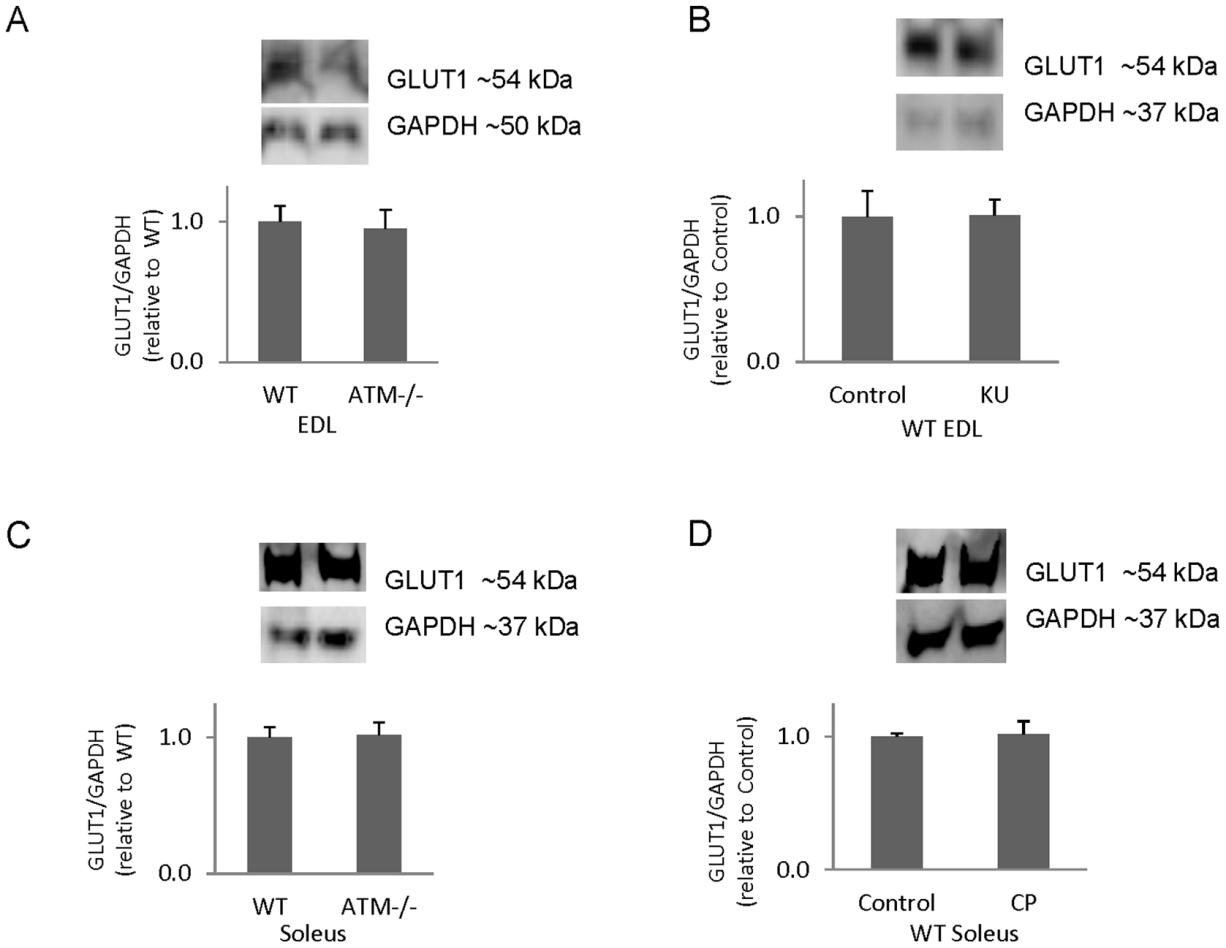
The effects of chronic ATM loss and ATM inhibition on GLUT1 protein levels. Mouse muscles were excised and subjected to western analysis for GLUT1 A) Wild type (WT) and ATM mutant (ATM −/−) EDL muscles (n = 3/group). B) WT EDL muscles plus/minus 10 µM KU (n = 6/group). C) WT and ATM −/− soleus muscles (n = 4/group). **D)** Wild type soleus muscles plus/minus 6 µM CP (n = 5/group). *p<0.05.

GLUT1 protein levels are unaltered in mouse skeletal muscles by ATM inhibition with KU or CP, and GLUT1 levels are not statistically different in ATM deficient mice compared to wild type mice (Fig. 6A-D). We have previously reported that skeletal muscle GLUT4 levels do not differ between wild-type and ATM-deficient mice in either soleus or EDL [41]. Thus, differences in basal transport in ATM-deficient muscles do not appear to be caused by changes in GLUT1 or GLUT4 abundance.

## Discussion

GLUT1 is generally considered to be localized to the plasma membrane and responsible for basal glucose uptake. In the present study, we demonstrate that GLUT1 plasma membrane localization and GLUT1-mediated glucose and DHA transport are regulated by GLUT1 serine 490 (S490) and the serine/threonine protein kinase, ATM.

The last four amino acids of the 492 residue GLUT1 protein form the PDZ binding motif (DSQV). By mutations of those four amino acids and the short hairpin RNA knock down of Gα-interacting protein-interacting protein, c-terminus (GIPC1), a protein found to interact with those four amino acids, one study discovered that the PDZ binding motif plays a role in the regulation of GLUT1 transport, trafficking, and protein levels via GIPC1 by promoting cell surface retention of GLUT1 and protecting it against lysosomal degradation [15]. GIPC1-deficient cells displayed a reduction in glucose transport and cell surface GLUT1 but no change in total GLUT1 protein levels [15]. Likewise, blocking the association of GIPC1 with GLUT1 by expression of truncated GLUT1 lacking the PDZ binding domain shifts GLUT1 localization toward intracellular vesicles in canine kidney epithelial cells [14]. In contrast, GLUT1 localization was normal for a form of GLUT1 with its PDZ binding domain replaced with a different PDZ binding domain that also interacts with GIPC1. GIPC1 has previously been implicated in recycling of endosomal proteins to the plasma membrane [42]. Here we show that ATM promotes increased GLUT1-mediated transport by increasing the GLUT1/GIPC1 association and increasing GLUT1 externalization.

The c-terminus of GLUT1, but not GLUT3 or GLUT4, interacts with GIPC1 [43]. GLUT4’s c-terminal sequence does not match consensus PDZ binding motifs as described by Stiffler et al [44]. On the other hand, the c-terminus of human GLUT3 (T-N-V) matches the class I PDZ binding motif (S/T-X-V/L), and the c-terminal of rat and mouse GLUT3 (G-N-A) correspond to a class III PDZ binding motif (ψ-X-ψ, where ψ is a hydrophobic amino acid). Still, there are no entries for GLUT3 or GLUT4 in PDZBase, a curated database for interactions involving PDZ domains (http://icb.med.cornell.edu/services/pdz/start) [45]. Thus, if there are functional PDZ binding sites in GLUT3 or GLUT4, these have not yet been discovered.

Truncation and/or alteration of the GLUT1 c-terminus has been shown to result in an internal localization [13]–[15]. GLUT1 S490, located in the c-terminal PDZ binding motif that interacts with GIPC1, is a known ATM phosphorylation target [22]. Here we show that GLUT1 S490 mutated to alanine reduces GLUT1-mediated transport and diminishes association of GLUT1 with GIPC1. Our findings are consistent with previous reports that ablation of the PDZ binding motif via S490A prevents interaction of GLUT1 with GIPC1 [14]. However, the current study extends this work by showing that activation of ATM, phosphorylation of GLUT1, or mimicking GLUT1-S490 phosphorylation with a S490D mutation strengthens the GLUT1/GIPC1 interaction. Based on the nomenclature of Stiffler et. al. [44], GLUT1 amino acid residues 490–492 (SQV) comprise a Class I PDZ binding motif (S/T-X-V/L, where X is any amino acid). Intriguingly, the S490D mutation changes the site to a Class III motif (E/D-X-ψ, where ψ is any hydrophobic amino acid), and phosphorylation of S490 would create this motif as well. There is a bit of promiscuity of PDZ binding domains, as some PDZ proteins that bind Class I binding partners can also bind Class III binding motifs [44]. Our findings suggest the possibility that alteration of the PDZ binding motif from a Class I motif to a Class III motif promotes association of GLUT1 with GIPC1.

Based on GLUT1 structure models [40], [46], [47], one of the GLUT1 S/T-Q motifs occurs in a transmembrane sequence and is not a candidate for phosphorylation by ATM. The remaining three are in cytoplasmic regions of GLUT1, and two of these (S490 and S473) are reportedly phosphorylated (PhosphoSitePlus®, www.phosphosite.org) [38]. In contrast, none of the S/T-Q motifs in GLUT4 have been reported to be phosphorylated. While T232 (TQ) of human GLUT3 has been reported to be phosphorylated, the sequence is different in mouse (TS) and rat (TP) and thus would not affect the L6 or mouse data shown.

It has been established that ATM plays a role in regulation of insulin-stimulated (i.e. predominantly GLUT4-mediated) glucose uptake in muscle cells and skeletal muscle. For example, expression of kinase-dead ATM prevents the insulin-stimulated increase of cell surface GLUT4 in L6 myoblasts [19]. This seems to be a result of a role of ATM in insulin-stimulated phosphorylation of Akt and/or AS160 [18], [19], [41], two key regulators of insulin-stimulated glucose transport. It seems unlikely, however, that there is direct action of ATM on GLUT4, as none of the ATM target motifs (S/T-Q) in GLUT4 have been reported to be phosphorylated (PhosphoSitePlus®, www.phosphosite.org, [38].

A recent study using Clone 9 cells, a rat liver cell with GLUT1 as its only GLUT, demonstrated that overexpression of murine and human stomatin, an integral membrane protein found in many tissues, decreased glucose transport [17]. We hypothesized that altered GLUT1/stomatin association would underlie changes in glucose and DHA transport, but this was not the case. Thus, changes in transport in the current study appear more likely to be attributable to changes in the GLUT1/GIPC1 interaction.

Interestingly, effects of ATM activation, ATM inhibition, and expression of GLUT site mutants affected transport to a greater degree than cell surface GLUT1. This suggests that ATM and GLUT1-S490 regulate GLUT1 intrinsic activity in addition to cell surface localization. Considering that acute ATM inhibition/activation is sufficient to cause regulatory effects on GLUT1 cell surface levels and GLUT1 transport, processes such as GLUT1 synthesis are unlikely to be the cause of the rapid regulatory effects seen here. GLUT1 protein degradation may be involved in these regulatory effects [48]. However, in apoptosis resistant Bak−/− Bax−/− cells internalized GLUT1 may reside internally for up to several weeks before being targeted for lysosomal degradation [49]. On the other hand, another study demonstrated a rapid lysosomal targeting of GLUT1 when the final four amino acids of GLUT1 (which include S490) were altered [15].

In haemopoietic cells, cytokines, interleukin-3 and -7 (IL-3 and IL-7), and growth factors regulate GLUT1 protein levels and cell surface appearance in an Akt-dependent fashion [26], [50]. In one study, increased Akt levels inhibited the internalization of GLUT1 [48]. Here, we show that ATM has no effect on the internalization rate of GLUT1 but rather plays a role in its externalization. Previous studies have shown that ATM plays a role in phosphorylation of Akt at S473 in Cos cells [51]. However, Akt phosphorylation is not regulated by ATM in L6 cells [18]. Thus, previous data and the current findings suggest that the trafficking of GLUT1 may be regulated by multiple pathways.

Understanding the roles and mechanisms of GLUT1 regulation has implications in several medically relevant areas. GLUT4 knockout mice, which were insulin resistant but not diabetic, displayed increased levels of cardiac GLUT1, suggesting that GLUT1 may compensate for lack of insulin-stimulated GLUT4-mediated transport [52]. Patients with type 2 diabetes display deficiencies in basal glucose transport and lowered GLUT1 protein levels in skeletal muscle [5]. Therefore, gaining a solid understanding of the factors that regulate basal glucose transport in skeletal muscle could point to novel approaches for improving glucose clearance in T2DM patients as a way of compensating for insulin resistance. Equally important, expanding knowledge on the mechanisms of augmented DHA transport has implications for insulin resistance as well. DHA is converted to ascorbic acid, which scavenges reactive oxygen species (ROS) [53]. Previous studies have shown that ROS are associated with insulin resistance in skeletal muscle [54]–[57]. Thus, understanding the underlying mechanisms of DHA transport may yield evidence as to how to combat ROS-related insulin resistance.

The present study examined several factors involved in the regulation of GLUT1 in cultured muscle and skeletal muscle. Three GLUT isoforms are expressed in L6 myoblasts: GLUT1, GLUT3, and GLUT4 [27], [58]. GLUT4 was inhibited with indinavir and the potential influence of GLUT3, although minimally expressed in L6 myoblasts [27], is likely to be negligible in the face of overexpression of GLUT1. In skeletal muscle, GLUT1, GLUT4, GLUT5, and GLUT12 are expressed [59]. GLUT5 primarily transports fructose with very low affinity for glucose [59]. GLUT12, although translocatable [60], has no known function in skeletal muscle [59]. Thus, the current study effectively focuses on the regulation of GLUT1 in muscle.

In summary, ATM and GLUT1-S490 appear to support GLUT1-mediated transport in skeletal muscle cells by promoting GLUT1 trafficking to the plasma membrane, associated with increased interaction of GLUT1 with GIPC1.
